# Using Artificial Neural Networks to Relate External Sensory Features to Internal Decisional Evidence

**DOI:** 10.1162/OPMI.a.317

**Published:** 2026-01-15

**Authors:** Marshall L. Green, Mingjia Hu, Rachel N. Denison, Dobromir Rahnev

**Affiliations:** School of Psychology, Georgia Institute of Technology, Atlanta, GA, USA; Department of Psychology, Indiana University, Bloomington, IN, USA; Department of Psychological and Brain Sciences, Boston University, Boston, MA, USA

**Keywords:** perceptual decision-making, orientation discrimination, convolutional neural network, signal detection

## Abstract

All theories of perceptual decision-making postulate that external sensory information is transformed into the internal evidence that is used to judge the identity of the stimulus. However, the nature of this external-to-internal transformation is generally unknown. In two experiments, we examined how a particular stimulus feature—orientation—is transformed into internal evidence. Subjects judged whether Gabors were tilted clockwise or counterclockwise. The results of Experiment 1 demonstrated that increasing the stimulus tilt in fine-scale increments resulted in a linear increase in sensitivity. However, the results of Experiment 2 demonstrated that increasing the stimulus tilt in coarse-scale increments had little effect on sensitivity, suggesting a highly non-linear transformation. Critically, artificial neural networks (ANNs) trained on the orientation task reproduced the empirical results, providing a framework for examining this external-to-internal transformation. The ANNs’ internal activations revealed that fine-scale increments in tilt magnitude results in increasingly greater discriminability between the stimulus categories, but the degree of discriminability does not increase further after tilt magnitude becomes sufficiently large. Taken together, these results begin to reveal how external sensory information is transformed into the internal evidence that is used to judge the identity of a stimulus and suggest that ANNs could serve as a platform for understanding the mechanism underlying this critical transformation.

## INTRODUCTION

**Figure 1.**
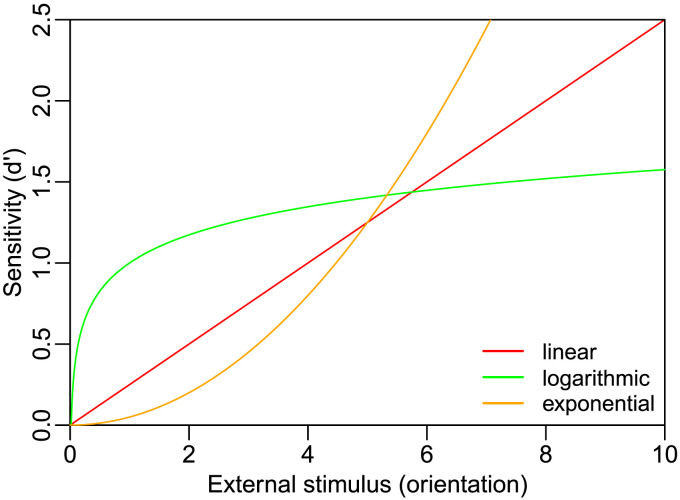
**Possible external-to-internal transformations**. Three possible transformations from orientation to task sensitivity (*d*′). The correct transformation is typically unknown.

Consider a decision-maker who discriminates between Gabor patches oriented one degree to the left versus right of vertical. According to signal detection theory (SDT), stimuli with left and right orientation are each transformed into internal evidence that is normally distributed along a horizontal decision axis. SDT allows us to compute the decision-maker’s sensitivity in this task (e.g., *d*′ = 1). However, SDT is silent regarding how the external stimulus feature (in this case orientation) relates to the strength of the internal signal (which we can measure as task sensitivity, *d*′). For example, what does the fact that *d*′ = 1 when discriminating between −1°/1° stimuli tell us about what *d*′ we would expect when discriminating between −2°/2° stimuli? The relationship of degrees tilt (*x* axis) and *d*′ (*y* axis) could be linear, logarithmic, or exponential (Figure 1). Thus, the exact nature of how an external feature (e.g., orientation) is transformed into internal evidence (e.g., quantified as sensitivity, *d*′) can take many forms and remains unclear.

Scientists have developed many models of perceptual decision making that can be used to understand the external-to-internal transformations. For example, one possible assumption is that a stimulus with a given orientation produces activations in a population of differently tuned neurons with the decision reflecting the preferred orientation of the neuron with the strongest response (i.e., winner-take-all; Lee et al., 1999). Another example is probabilistic population coding (PPC) models, which also consider a population of differently tuned neurons but make decisions based on a process of Bayesian inference by considering the activations across the whole population (Ma et al., 2006). However, these models generally do not allow us to determine a priori how different manipulations (e.g., orientation magnitude or stimulus noise) affect the population response and therefore it can be difficult to use them to generate predictions for previously untested stimuli.

In this paper we test whether artificial neural networks (ANNs) can be used to make such a priori predictions. ANNs are a powerful way to model human behavior (Doerig et al., 2023; Kriegeskorte, 2015; Ma & Peters, 2020) and the leading class of models of the mechanisms underlying primate vision (Kubilius et al., 2019). The strength of ANNs comes from the fact that they can be trained on images (most other models in cognitive science and computational neuroscience do not work on images). By training ANNs to classify large sets of visual stimuli, they can learn patterns and features that are difficult to intuit or build into traditional models. This atheoretical learning process makes them particularly useful for discovering how sensory features map onto internal evidence because the external-to-internal mapping is emergent.

To examine whether ANNs can capture the external-to-internal transformation, we examine how a particular stimulus feature—orientation—is transformed into internal decision evidence. Experiment 1 demonstrated that in a high-contrast fine-discrimination task, orientation is linearly transformed into internal evidence, such that stimulus sensitivity (*d*′) increases proportionately to orientation. However, Experiment 2 showed that a noisy low-contrast coarse-discrimination task produces a very different relationship with an initial increase in *d*′ followed by a plateau. Critically, we demonstrate that ANNs trained on orientation categorization, mirrored the empirical results, thus providing a framework for examining how the sensory stimuli map onto internal decisional evidence. These results were explained by examining how orientation affected the activation distributions of the ANNs’ output layer. These results suggest that external sensory information is transformed into internal decisional evidence in complex ways that may not be easily intuited from fitting traditional models and demonstrate that ANNs could serve as a modeling platform for understanding the mechanism underlying this critical transformation.

## METHODS

### Participants

A set of 13 subjects were recruited for Experiment 1 and a separate set of 12 subjects were recruited for Experiment 2 (25 subjects total). A power analysis was not conducted prior to data collection, and the data sample for each experiment was obtained out of convenience. Post hoc power analyses showed that sufficient power (>.9) was obtained in both experiments given the variance explained by the preferred models (*R*^2^ > .76) and the number of subjects included in analyses (see Supplementary for details). All subjects had normal or corrected to normal vision. One subject in Experiment 1 and one subject in Experiment 2 were excluded from analyses due to chance performance (50% accuracy across all conditions). All subjects provided informed consent and were compensated for their participation. All research methods and protocols were approved by the Georgia Institute of Technology Institutional Review Board and were performed in accordance with relevant guidelines and regulations.

### Task and Stimuli

**Figure 2.**
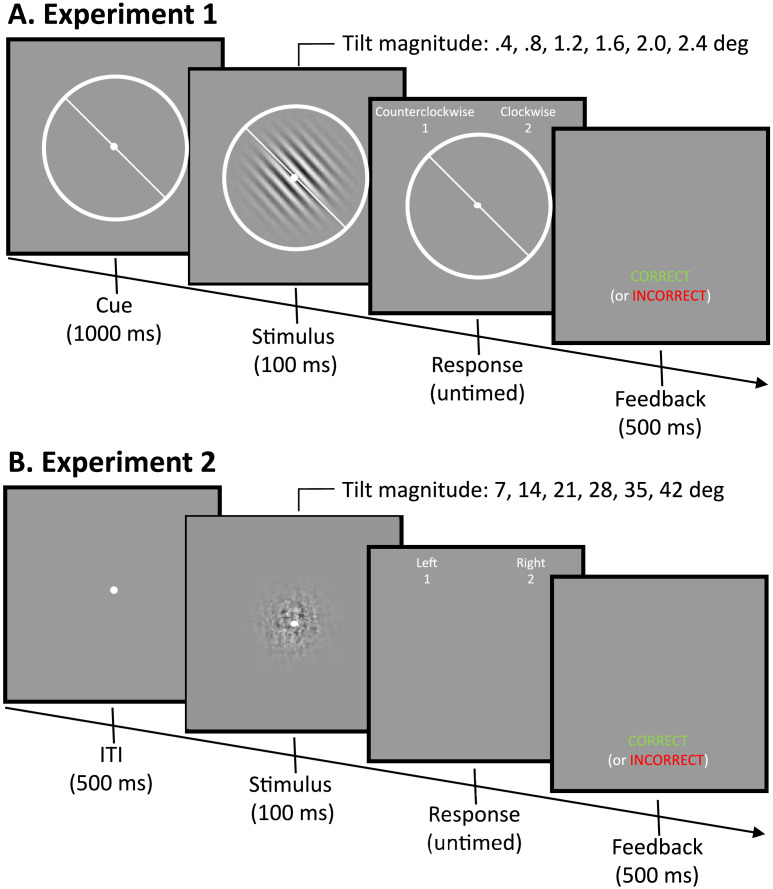
**Tasks for Experiments 1 and 2.** (A) Experiment 1. Subjects judged whether high-contrast Gabor patches were tilted clockwise or counterclockwise from a 45-degree cue. The Gabor patches varied in fine-scale increments from .4 to 2.4 degrees. (B) Experiment 2. Subjects judged whether noisy, low-contrast Gabor patches were tilted clockwise or counterclockwise from vertical. The Gabor patches varied in coarse-scale increments from 7 to 42 degrees.

In Experiment 1, subjects performed a fine-scale orientation categorization task in which a Gabor stimulus was tilted counterclockwise or clockwise of 45° (Figure 2A). On each trial, subjects fixated on a small white dot presented at the display center. A visual cue consisting of a circle (diameter = 4.5° visual angle) and a line (length = 4.5° visual angle) oriented at 45° was presented for 1000 ms. Following fixation and cueing, a Gabor patch (diameter = 4° visual angle) with a frequency of 3 cycles per degree visual angle was presented for 100 ms at full contrast. Immediately after the stimulus presentation subjects provided their response by pressing “1” to respond “counterclockwise” or “2” to respond “clockwise”. After a response was made the subject was given accuracy feedback for 500 ms.

In Experiment 2, subjects performed a coarse-scale orientation categorization task in which a Gabor stimulus was tilted left (counterclockwise) or right (clockwise) of vertical (Figure 2B). On each trial, subjects fixated on a small white dot presented at the display center for 500 ms. Following fixation, a Gabor patch (diameter = 4° visual angle) with a frequency of 3 cycles per degree visual angle was presented for 100 ms at 9% contrast embedded in random pixel noise at 90% contrast. Noise was included to prevent ceiling performance, and the level of noise was selected to keep performance in a range that is between chance level (50%) and perfect performance (100%). We selected these combinations of stimulus and noise contrast based on a previous study (Bang et al., 2019) and confirmed that they lead to a reasonable range of performance values in pilot testing. Immediately after the stimulus presentation, subjects provided their response by pressing “1” to respond “left” or “2” to respond “right”. After a response was made the subject was given accuracy feedback for 500 ms. Responses were not speeded, and participants were given unlimited time to respond in both experiments. Participants were instructed to make accurate decisions in both experiments.

### Procedure

In Experiment 1, Gabor stimuli were tilted away from 45° and the magnitude of tilt was manipulated across six levels (.4°, .8°, 1.2°, 1.6°, 2°, and 2.4°) that were randomly chosen on each trial and counterbalanced across counterclockwise and clockwise directions. Prior to beginning the experimental runs, subjects received task instructions and then completed practice trials consisting of 20 easy trials with a 10° tilt, 40 moderately difficult trials (10 each at 5°, 3°, 2°, and .5°), and finally 20 trials with a 1.4° tilt. Subjects completed four experimental runs each consisting of six 45-trial blocks for a total of 1,080 trials such that each tilt condition had an equal number of 180 trials.

In Experiment 2, Gabor stimuli were tilted away from vertical, and the magnitude of stimulus tilt was manipulated across five levels (7°, 14°, 21°, 28°, 35°, and 42°) which were randomly chosen on each trial and counterbalanced across left and right directions. Prior to beginning experimental runs, subjects received task instructions and then performed practice trials consisting of 20 easy trials with a 42° tilt at 30% contrast, 40 moderately difficult trials at decreasing tilts 35°, 28°, 14°, and 7° (10 trials each) and at 35%, 28%, 10% and .9% contrast respectively, and finally 30 trials with a 21° tilt at decreasing contrasts (12%, 10%, and 9%, 10 trials each). Subjects completed four experimental runs each consisting of six 45-trial blocks for a total of 1,080 trials such that each tilt condition had an equal number of 180 trials.

### Apparatus

Both Experiments 1 and 2 were designed in the MATLAB environment using Psychtoolbox 3 (Brainard, 1997). Stimuli were presented on a 21.5-inch iMac monitor (1920 × 1080 pixel resolution, 60 Hz refresh rate) in a dark room. Subjects were seated 60 cm away from the display and provided their responses using a standard computer keyboard.

### Analysis

To compare subjects’ sensitivity to orientation across conditions, we computed *d*′ using the standard formula (Green & Swets, 1966) by treating clockwise tilt trials as the target and calculating the hit rate (HR) and false alarm rate (FAR):

$$d^{\prime }=\Phi^{-1}\left(HR\right)-\Phi^{-1}\left(FAR\right)$$

where Φ^−1^ is the inverse of the cumulative standard normal distribution that transforms the HR and FAR into *z*-scores. Sensitivity (*d*′) was computed separately for each individual and each condition. This same approach was used to compute sensitivity (*d*′) from the HR and FAR produced by the ANN model.

We fit a series of random effects regression models to estimate the function that best describes how external sensory signal strength—the magnitude of stimulus tilt—maps onto internal evidence as measured with sensitivity (*d*′) for each individual. A random effects model allows each individual to have their own unique regression relationship by estimating both the random slope and random intercept. A model which describes a linear relationship between the magnitude of stimulus tilt and sensitivity is given as:

$$d^{\prime }=\alpha +\beta X+\varepsilon$$

where *α* is the intercept, *β* is the weight of the linear slope, and *ε* is random error.

Neither Experiment 1 nor 2 included a condition in which the stimulus was not tilted at all since such a condition would not have a correct decision. However, we assumed a hypothetical sensitivity (*d*′) of zero by constraining the intercept to the origin. By fixing the intercept to be zero, the intercept term can be dropped, and the linear model can be reduced to:

$$d^{\prime }=\beta X+\varepsilon$$

To account for potential nonlinear external-to-internal mappings, we fit several additional models. First, we fit a quadratic regression model whereby the effect of manipulating the magnitude of stimulus tilt on sensitivity (*d*′) is given as:

$$d^{\prime }=\beta_{1}X+\beta_{2}X^{2}+\varepsilon$$

We then fit a polynomial regression model by including an additional exponent term of the third degree whereby the effect of manipulating tilt magnitude on sensitivity is given as:

$$d^{\prime }=\beta_{1}X+\beta_{2}X^{2}+\beta_{3}X^{3}+\varepsilon$$

Finally, we fit a logarithmic model whereby the effect of manipulating tilt magnitude on sensitivity is given as a combination of a linear and a logarithmic components:

$$d^{\prime }=\beta_{1}X+\beta_{2}\text{logX}+\varepsilon$$

To assess which regression model best describes the transformation of external sensory signals into internal evidence, we used Bayesian Information Criterion (BIC; Schwarz, 1978) to compare the relative fits of these regression models to each other. BIC penalizes model complexity by taking the product of the number of parameters and the natural log of the number of data points. Similar results are found if models are compared using Akaike’s information criterion (AIC; Akaike, 1973) which is more lenient on model complexity than BIC because the penalty term is a constant factor of two.

### Artificial Neural Network (ANN) Models

**Figure 3.**
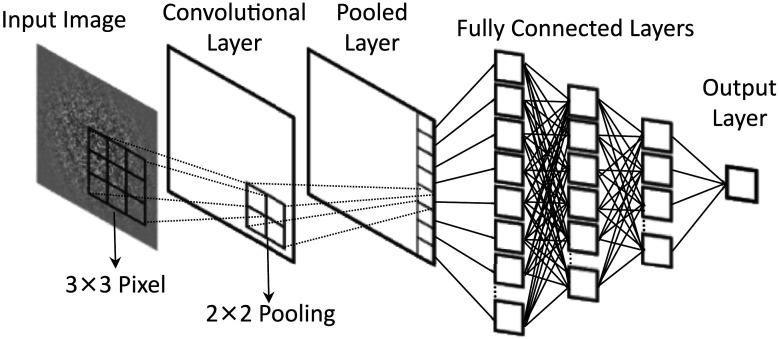
**Schematic of the ANN models.** We tested 3-layer, 4-layer, and 5-layer ANNs. All ANN models take as input a 100 × 100 image and outputs one of two category labels corresponding to counterclockwise (left) or clockwise (right) orientation. The models consist of one convolutional layer, one pooling layer, and between one and three fully connected layers. The convolutional layer consists of a set of one linear filter with equally sized receptive fields (e.g., 3 × 3 pixels) with equally spaced intervals followed by rectified linear unit (ReLu) activation. The output of the convolutional layer is pooled to reduce the size of the input volume by taking the maximum activation value of 2 × 2 spatially localized units. This pooled result was then flattened and passed to the fully connected layers followed by a decision unit. The graph depicts the 5-layer ANN.

We built three simple feedforward ANN models (Figure 3) using the TensorFlow toolbox (Abadi et al., 2016). The models take as input a 100 × 100 image and output one of two category labels corresponding to clockwise or counterclockwise tilt. The models consist of one convolutional layer, one pooling layer, and between one and three fully connected hidden layers. The convolutional layer consists of a linear filter with equally sized receptive fields (3 × 3 pixels) and with equally spaced intervals followed by rectified linear unit (ReLU) activation function. This processing operation results in a 98 × 98 activation map encoding the response of the filter at each spatially localized region of an image. The output of the convolutional layer is pooled to reduce the size of the input volume by taking the maximum activation value of 2 × 2 spatially localized units resulting in a 49 × 49 output volume. This pooled result was then flattened and passed to the fully connected layers followed by a decision unit. The 5-layer model which, in total, consists of a convolutional layer (plus pooling layer), 3 hidden layers with 256, 128, and 64 units across descending layers, and 1 output layer, has a total of 656,139 trainable weights. In addition to this 5-layer model, a 4-layer and a 3-layer model were created by removing either the last fully connected layer or the last two fully connected layers for the purpose of examining the effect of model depth on performance.

The ANN models were trained to categorize Gabor stimuli as tilted counterclockwise or clockwise of vertical. The total training set consisted of 10,000 stimuli. During training, the degree of tilt, contrast, and noise of each Gabor stimulus was randomly drawn from uniform distributions. The sampling distribution for tilt magnitude ranged from 0 to 60 degrees, for contrast from 1% to 90% (i.e., amplitude from .01 to .9) and for noise from 1% to 100%. The visual noise was created by embedding random pixel noise into the Gabor stimulus. The midpoint gray of the stimulus was defined as 0 with black being −1 and white being 1. When combining pixel values for the Gabor and noise, it is possible that some pixels will exceed the intended range and result in a stimulus contrast greater than 100%, particularly when both contrast and noise are high. To avoid this issue, pixel values less than −1 were set equal to −1, and pixels greater than 1 were set equal to 1, ensuring that the total stimulus contrast was within the range of 0 to 100%. This correction mostly applied to stimuli with high contrast and high noise and was at maximum applied to 1.6% of the pixels (for the stimuli with 100% noise and .9 contrast), which did not substantially affect the visual appearance of these stimuli. Learning was evaluated by testing the model on 1,000 novel Gabor stimuli generated from the same sampling distributions for tilt, contrast, and noise. To reduce the chance of idiosyncratic model behavior due to the random starting weights, we trained 30 model initializations of each model and averaged their results. The models were trained for 10 epochs.

The trained ANNs were then tested on fine and coarse-scale orientation categorization tasks. For the fine-scale orientation categorization task, orientation offset was varied across 29 equally spaced levels ranging from 0 to 2.8 degrees. For the coarse-scale orientation categorization task, orientation offset was varied across 29 equally spaced levels ranging from 0 to 49 degrees. For both the fine- and coarse-scale tests of the ANNs, the Gabor stimuli varied in contrast across five levels ranging from 2.5% to 8.5%, and visual noise varied in contrast across four levels ranging from 25% to 100%. As with the stimuli in the training set, we set pixels less than −1 to −1, and pixels greater than 1 to 1 (note that this was only necessary for the 100% noise condition). The ANNs were tested on 1,000 stimuli at each combination of tilt and stimulus category (clockwise or counterclockwise tilt), resulting in a total of 58,000 simulated trials for each task. To reduce the chance of idiosyncratic model behavior due to the random starting weights, we trained 30 model initializations of each ANN and averaged their results.

We then analyzed the linear activation output of the models’ penultimate layer in response to the Gabor stimuli for each orientation category and for each tilt magnitude condition (Shekhar & Rahnev, 2024). These activation distributions represent the internal evidence for each stimulus category (clockwise and counterclockwise) and provide a way to evaluate how the internal evidence of the ANN is affected by increasing the magnitude of tilt. We then quantified the discriminability of the two stimulus categories as the distance between the means of the internal evidence distributions, and we quantified their noisiness as the standard deviation of the two evidence distributions. The means and standard deviations of the evidence distributions were computed separately for each of the 30 instances of each model. The internal activation distributions were computed from the linear outputs rather than standardized (z) units; however, dividing the mean difference by the pooled standard deviation is theoretically analogous to computing the means and standard deviation of signal detection information. Consistent results are obtained when the linear activations are first z-transformed and separability is computed in the standardized space. Moreover, this analysis yields results highly similar to those using the conventional signal detection measure applied to the model’s hit rate (HR) and false alarm rate (FAR) produces.

We also analyzed the representational similarity (Diedrichsen & Kriegeskorte, 2017) of the final fully connected layer of each model to examine whether the models were representing the stimuli in a manner consistent with the categorization task (see Supplementary for details).

## RESULTS

Our goal was to examine how external sensory information is transformed into internal decisional evidence. In two behavioral experiments, we empirically tested how a particular stimulus feature—orientation—is transformed into internal evidence for similar decisions with different task contexts. We tested ANNs trained on similar tasks and found that they reproduced the empirical results. We then examined the ANNs’ internal representations to assess the mechanism underlying this critical transformation.

### Behavioral Results

**Figure 4.**
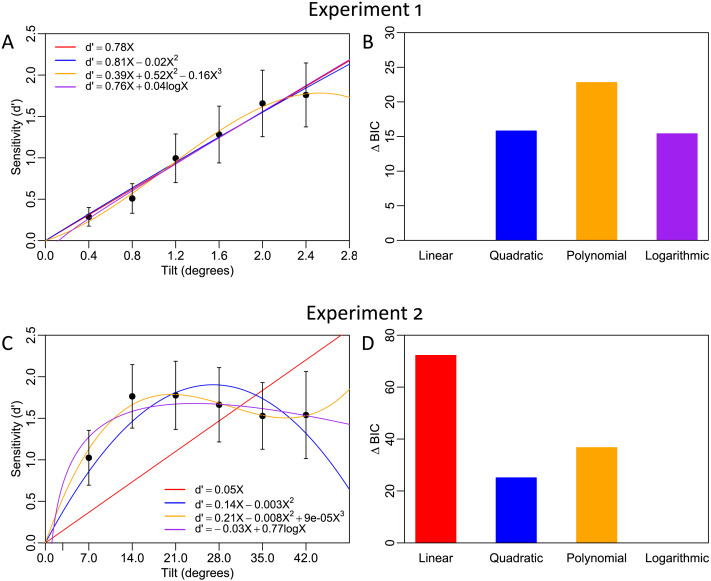
**Behavioral results**. (A) Experiment 1 results. Increasing the magnitude of tilt in fine-scale increments results in a linear increase in sensitivity (*d*′). (B) Model comparison for Experiment 1. A model comparison using BIC demonstrates that the linear model with the intercept constrained to the origin (red line) was the best performing model, suggesting a linear external-to-internal transformation. (C) Experiment 2 results. Sufficiently large increases in tilt magnitude had little effect on sensitivity. (D) Model comparison for Experiment 2. BIC suggested that the logarithmic model (purple line) was the best performing model, suggesting a highly non-linear transformation. Error bars in panels (A) and (C) show 95% confidence intervals; lines show best fitting functions.

In Experiment 1, subjects judged the orientation of a high-contrast Gabor that was tilted in fine-scale increments from .4 to 2.4 degrees away from a 45-degree cue (Figure 2A). We found that sensitivity (*d*′) appears to increase linearly as the magnitude of tilt offset was increased (Figure 4A). To examine the linearity of the function, we fit random effects regression models to characterize how sensitivity (*d*′) varied as a function of tilt magnitude. We found that a slope-only model with the intercept constrained to zero provides the best fit (*d*′ = .78*x*, *t*(60) = 8.70, *p* < .001). A linear model that includes the intercept did not meaningfully improve the model fit (Δ*BIC* = 15.93, Δ*AIC* = 9.23; model: *d*′ = −.03 + .79*x*) because, although the linear slope parameter was significant (*t*(59) = 7.87, *p* < .001), the intercept was not (*t*(59) = −.38, *p* = .70). Similarly, neither the inclusion of a quadratic term (Δ*BIC* = 15.80, *AIC* = 9.09, *t*(59) = −.37, *p* = .71; Figure 4B), third-degree polynomial term (Δ*BIC* = 22.80, Δ*AIC* = 7.25, *t*(58) = −1.80, *p* = .08), nor logarithmic term (Δ*BIC* = 15.40, Δ*AIC* = 8.69, *t*(59) = .63, *p* = .53) outperformed the constrained linear model. Although each of the models can reasonably capture the data, suggesting that these models are not well differentiated by the fine-scale task, the linear model with the intercept constrained to zero provides the most parsimonious fit to the data and including additional nonlinear parameters did not improve the model. Altogether, this pattern of results suggests that orientation is linearly transformed into internal decision evidence in a fine-scale discrimination task.

Critically, we found a strikingly different pattern of results for Experiment 2 where subjects judged the orientation of a noisy, low-contrast Gabor in coarse-scale increments from 7 to 42 degrees away from vertical (Figure 2B). Instead of sensitivity (*d*′) linearly increasing with the magnitude of tilt, we found that above 14 degrees tilt sensitivity no longer increased and appeared to slightly decrease (Figure 4C). This effect was not due to either floor or ceiling effects as the accuracy in the different conditions was in a range (69–79%) that is far from both chance level (50%) or perfect performance (100%).

In stark contrast with the results of Experiment 1, in Experiment 2, the linear model with the slope constrained to zero provided for a subjectively poor fit to the data (Figure 4C, red line). Despite this poor fit to the data, the linear slope parameter of the constrained linear model is significant (*t*(55) = 7.62, *p* < .001; model: *d*′ = .05*x*). But including the intercept in the linear model (*d*′ = 1.37 + .007*x*, *t*(54) = 8.64, *p* < .001) provides for a better fit relative than the constrained linear model (Δ*BIC* = 55.90, Δ*AIC* = 62.33) and demonstrates that the linear slope parameter is a poor predictor of the outcome (*t*(54) = 1.31, *p* = .19). However, it is crucial to focus on a model in which the intercept is constrained to zero because, in theory, sensitivity (*d*′) should approach zero as the external signal becomes sufficiently weak. The full model comparison analysis revealed that the logarithmic model (*d*′ = −.03*x* + .77 log(*x*)) outperformed the slope-only linear model (Δ*BIC* = 72.22, Δ*AIC* = 78.65), the full linear model (Δ*BIC* = 16.32, Δ*AIC* = 16.32), the quadratic model (Δ*BIC* = 25.07, Δ*AIC* = 25.07), and the polynomial model (Δ*BIC* = 36.71, Δ*AIC* = 28.23). Both the linear term (*t*(54) = −4.62, *p* < .001) and logarithmic term (*t*(54) = 9.25, *p* < .001) of the logarithmic model were significant predictors of the outcome variable. The preferred logarithmic model (Figure 4D) features a steep initial increase followed by a mostly flat but slightly decreasing portion (Figure 4C, purple line) because the linear term was estimated to be negative. However, a purely logarithmic model would not predict this slight decrease because the logarithm, for all bases, is a monotonically increasing function. A follow-up comparison revealed that the model which includes both the linear and logarithmic terms was preferred over a purely logarithmic model (Δ*BIC* = 2.37, Δ*AIC* = 8.79; model: *d*′ = .49 log(*x*)) confirming that sensitivity begins to slightly decrease as the magnitude of tilt becomes sufficiently large. Taken together, these results reveal that, under the task context of Experiment 2, orientation is strongly nonlinearly transformed into internal decision evidence.

Here we applied signal detection theory to examine how the magnitude of tilt offset is transformed into the internal decisional evidence for identifying left or right orientation, and the results showed clear differences in this transformation across the tasks in Experiments 1 and 2. Although signal detection theory is a standard approach for analyzing data from two choice tasks, a complementary analytical approach is to plot the data as a rate of choosing one stimulus category and fit a sigmoid function. As expected, our data are indeed consistent with a sigmoid-shaped function when plotted in this manner (Figure S1). However, our results cannot be summarized as simply showing sigmoid functions because any reasonable relationship between the *rate of choosing one stimulus category* and tilt would result in a sigmoid function regardless of the exact relationship between *d*′ and tilt. Instead, working in the space of *d*′ vs. tilt is more informative for the goal of describing how external sensory information is transformed into the internal evidence used to identify a stimulus.

### ANN Results

The results from Experiments 1 and 2 show that intuitively similar external stimulus features can have different mappings to internal evidence depending on the task context and the range of stimulus features tested. While these results are likely to be explainable within different modeling frameworks, it is not clear whether any model frameworks would have predicted them a priori without additional assumptions about perceptual similarity functions. For example, an off-the-shelf probabilistic population coding model did not predict these results (Figure S2), though it is of course likely that a better fit can be obtained if additional assumptions were included in the model. Instead of attempting to make such additional assumptions about the nature of the external-to-internal mapping, here we use ANN models to investigate the transformation of external sensory stimuli to internal evidence.

**Figure 5.**
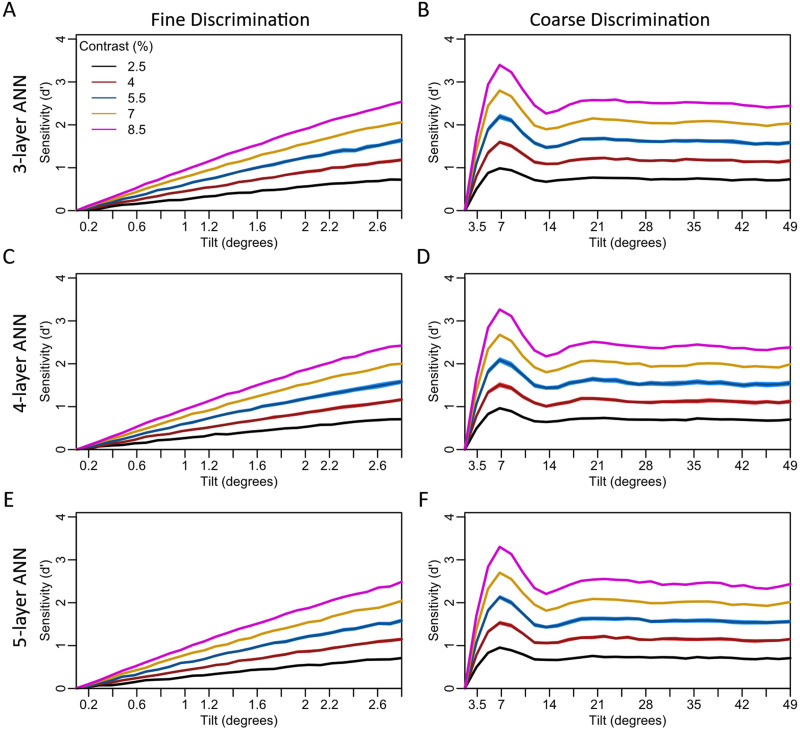
**Artificial neural network (ANN) results.** The ANNs results (lines) reproduced the empirical results from Experiment 1 and Experiment 2—fine-scale increments in the tilt magnitude from 0 to 2.8 degrees were linearly transformed into internal evidence (A, C, E), but coarse-scale increments in the tilt magnitude up to 49 degrees were nonlinearly transformed into internal evidence (B, D, F). The performance of the 3-layer (A, B), 4-layer (C, D) and 5-layer (E, F) models was nearly identical. For both tasks, increasing the stimulus contrast had a scaling-up effect on sensitivity but had little effect on the general trend in the relationship between sensitivity and tilt magnitude. The shaded regions show 95% confidence intervals, but they are difficult to see in the figure due to the small intervals and the Y-axis range.

We first evaluated whether ANN models trained on orientation categorization would naturally reproduce the observed external-to-internal transformations found in Experiments 1 and 2 without any additional assumptions or training. We trained 3-, 4-, and 5-layer ANN models on discriminating between Gabor patches tilted clockwise or counterclockwise from vertical. To provide an unbiased training set, we trained the ANNs on a wide range of tilts (0 to 60 degrees) and contrasts (1 to 90%). We then tested the trained ANNs on Gabor stimuli at contrasts ranging from 2.5 to 8.5 and at tilt magnitudes that mimic Experiment 1 (fine-grained tilts up to 2.8 degrees) and Experiment 2 (coarse-grained tilts up to 49 degrees); in both cases the noise level was set to 100%. We found that all three ANN models reproduced both the linear relationship between sensitivity and orientation for fine-grained tilts that we observed in Experiment 1 (Figure 5A, C, E), and the non-linear, flat relationship between sensitivity and orientation for coarse-grained tilts that we observed in Experiment 2 (Figure 5B, D, F). The depth (number of layers) of the ANN had no effect on the overall pattern as each ANN performed nearly equivalently even though a different set of stimuli were used to test each model and model instance. Nevertheless, unlike the human data in Experiment 2 where *d*′ peaks around 14 degrees, the ANN models have a maximum *d*′ around 7 degrees (see Discussion). Further, equivalent results were obtained when using noise levels of 25, 50, and 75% (Figure S3). Overall, these results suggest that the ANN model shows very similar emergent behavior to what we see in humans for the transformation from external sensory features to internal decision evidence, and that this general pattern of emergent behavior is robust to both manipulations of stimulus contrast and to model depth.

This pattern of results suggest that the ANN models show very similar emergent behavior to what we see in humans for the transformation from external sensory features to internal decision evidence. To understand the ANNs behavior, we examined the internal activations of the models. Here we focus our analysis on the internal behavior of the 3-layer ANN models because performance was unaffected by network depth and because it is the simplest of the three ANN models. Specifically, we analyzed the models’ output layer activation in response to the Gabor stimuli for each orientation category and for each tilt magnitude condition, providing a way to evaluate how the internal evidence of the ANNs is affected by increasing the magnitude of tilt.

**Figure 6.**
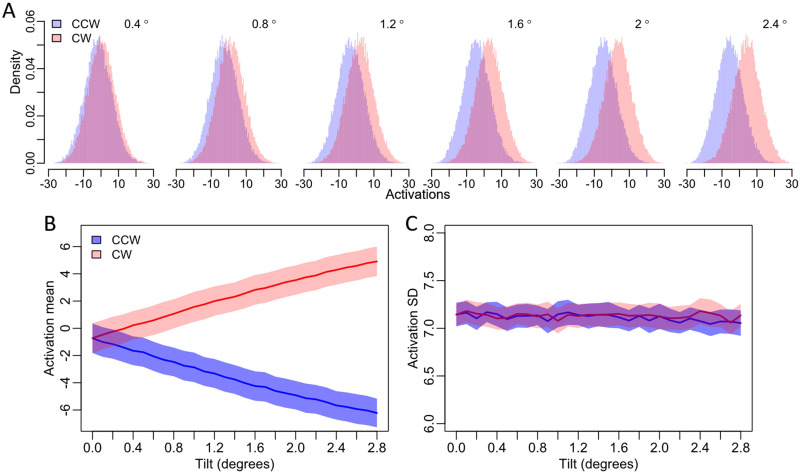
**ANN activations from small increases in tilt magnitude**. (A) Small, fine-scale increments in tilt magnitude result in systematic differences between the distributions of ANN activations in response to stimuli from each stimulus category (counterclockwise and clockwise). (B) The distribution means increase linearly. (C) However, the standard deviations of these distributions were entirely unaffected by tilt magnitude. The shaded regions in (B) and (C) show 95% confidence intervals.

We generated the internal evidence distributions for each orientation category for each tilt magnitude condition. We found that fine scale increases in tilt magnitude resulted in systematic differences between the evidence distributions for clockwise and counterclockwise stimulus categories. In particular, larger tilt offsets shifted the means of the distributions away from zero and away from each other but did not change the spread of evidence within each distribution (Figure 6A). Indeed, tilt magnitude had a large effect on the means of the distributions (Figure 6B, *F*(28, 812) = 1795, *p* < .001), but no effect on the standard deviation of the distributions for either stimulus category (Figure 6C, *F*s ≤ 1.48, *p*s ≥ .05). This systematic increase in the distance between the two evidence distributions with no change in standard deviation provides a causal explanation for why the ANN’s sensitivity (*d*′) increased linearly with increases in tilt magnitude.

**Figure 7.**
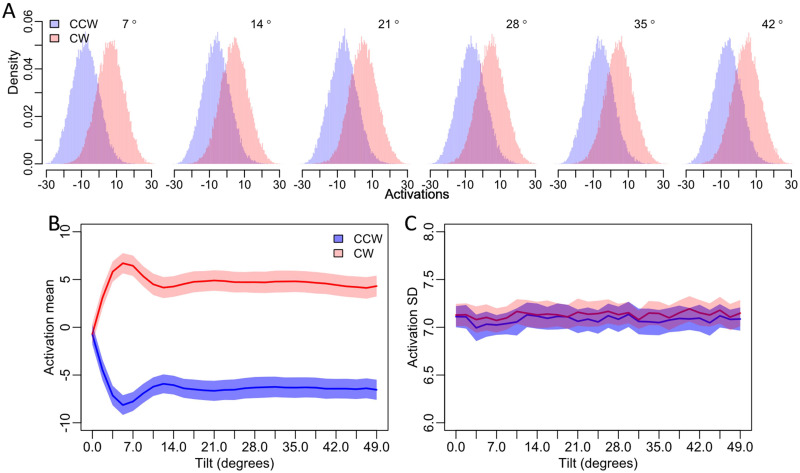
**ANN activations from large increases in tilt magnitude.** (A) Large, coarse-scale increments in tilt magnitude result in systematic nonlinear differences between the distributions of ANN activations in response to stimuli from each stimulus category (counterclockwise and clockwise). (B) The distribution means initially increase systematically before decreasing, plateauing, and then decreasing again. (C) The standard deviations of these distributions were unaffected by tilt magnitude. The shaded regions in (B) and (C) show 95% confidence intervals.

We conducted the same analyses using tilt offset across a larger range, thus mimicking the design of Experiment 2. We found that the coarse-scale manipulation of tilt magnitude had a significant but more complex effect on the means of the evidence distributions (*F*(28, 812) = 577.6, *p* < .001; Figure 7A), but again had no influence on the standard deviation of the distributions (Figure 7C, *F*s ≤ 1.48, *p*s > .05). In particular, at smaller tilt magnitudes between 0 and 5 degrees, the difference between the means of the evidence distributions systematically increased before peaking around 6.5 degrees, decreasing, and then plateauing between 14 and 49 degrees (Figure 6B). Taken together, these results suggest that the highly nonlinear relationship between orientation and sensitivity (*d*′) is driven by complex changes in the means of the internal evidence distributions but not the standard deviation of these distributions.

Lastly, we conducted a representational similarity analysis (RSA) to ensure that the ANNs were representing the orientation stimuli in a manner consistent with the task demands. The RSA results confirmed that the ANNs categorize left and right tilted stimuli as expected; there were greater correlations between all stimuli within a category than between categories (Figure S4). Moreover, we found that small increases in tilt led to a graded decrease in similarly. These results are congruent with our SDT-like analysis on the linear output from the final layer in which signal and noise distributions were more similar leading to less discriminability. However, the RSA results also revealed a robust increase in representational similarity for tilts beyond 7 degrees. This pattern is consistent with our finding that discriminability plateaus for sufficiently large tilts.

## DISCUSSION

We examined how external sensory information is transformed into internal decisional evidence across different task contexts. In Experiment 1, increasing the tilt of a high-contrast Gabor in fine-scale increments away from 45 degrees resulted in a linear increase in sensitivity (*d*′), suggesting a linear transformation from orientation to internal evidence strength. In contrast, in Experiment 2, increasing the tilt of a noisy, low-contrast Gabor in coarse-scale increments away from vertical resulted in a fast initial gain in sensitivity followed by a plateau with a slight decrease, suggesting a highly non-linear relationship between orientation and internal evidence strength. These results suggest that external sensory information is transformed into internal decisional evidence in complex ways that may not be clear from a snapshot of results obtained from a given experimental task. Critically, artificial neural networks (ANNs) trained on orientation categorization naturally reproduced this general pattern of results. Mechanistically, we discovered that when the stimulus tilt was increased in fine-scale increments, the ANNs’ internal distributions of evidence for each stimulus condition became more discriminable, but the degree of discriminability stopped increasing after sufficiently large and coarse increases in tilt magnitude. Taken together, these results begin to reveal how external sensory information is transformed into the internal decisional evidence and suggest that ANNs could serve as a platform for understanding the mechanism underlying this critical transformation.

### Dissociation Between Tasks Using Fine- and Coarse-Scale Stimuli

The general intuition of most theories of perceptual decision-making is that increasing the strength of a given external signal leads to a graded increase in the strength of the internal signal (e.g., Green & Swets, 1966; Ma et al., 2006; Ratcliff, 1978). In contrast with this intuition, we show that in the task with coarse-scale stimuli (Experiment 2) a large increase of the external signal does not translate into a monotonic one-to-one increase in the internal signal. Crucially, this pattern does not appear to reflect a ceiling or a floor effect because performance saturates in a range between 69 and 79% accuracy. What explains this pattern of results? There appear to be at least three different explanations.

First, it could be that performance differences across fine- and coarse-scale discrimination tasks are explained by the optimal readout mechanisms from feature-selective neurons (Britten et al., 1992; Celebrini & Newsome, 1995; Salzman et al., 1990, 1992). For fine-scale tasks highly similar stimulus features activate roughly the same population of feature selective neurons and so the most informative neurons are those tuned slightly away from the feature to be discriminated (Britten et al., 1992; Celebrini & Newsome, 1995; Salzman et al., 1990, 1992). But for coarse-scale tasks the most informative neurons are those tuned to the to-be-discriminated feature because the neurons which are selective for the competing stimulus category are less likely to be active, and as such all of the active neurons can be pooled for behavior (Shadlen & Newsome, 1996). Although these mechanisms are likely involved in fine and coarse orientation categorization tasks, they do not directly explain why performance plateaus, and even slightly decreases, in the coarse-scale task. Second, the coarse-scale task may involve threshold mechanisms which are not present in the fine-scale tasks; specifically that for the noisy Gabor patches in the coarse-scale task, there is some intensity of the external feature needed to perceive the stimulus (Rouder & Morey, 2009), and that when below this threshold participants are in a non-perceive state and make a guessing response (Green & Pratte, 2022). However, although such patterns in performance are typically modeled with a lapse rate, the high lapse rates implied in the coarse-scale task (>20%) are not normal, and the mechanism is different (as also shown by the equivalent ANN results, even though ANNs don’t lapse). Third, rather than stemming from different decisional or threshold mechanisms, it may simply be that any external feature is transformed into internal decisional evidence in complex ways that depend on a host of factors and are difficult to intuit.

### Using ANNs as Platforms for Evaluating the External-to-Internal Mapping

The typical approach to studying how external sensory information is transformed into internal decisional evidence is to use modeling frameworks like signal detection theory (SDT) to characterize the relationship between external sensory features and behavioral decisions. Although SDT is considered a standard analytical approach in studies of perception, one downside is that SDT provides only relative information about the signal and noise in the system and so it is ambiguous as to whether changes in performance result from changes in signal or noise (Denison et al., 2018; Rahnev et al., 2011). ANNs allow us to peer inside the system and directly examine how both signal and noise affect performance. We took the ANNs hidden-layer activations in response to stimuli of varying tilt magnitudes and applied an SDT-like analysis to the activation distributions (Shekhar & Rahnev, 2024). This approach provided a powerful way to directly examine the discriminability between signal and noise distributions, as well as the variability in the distributions. The ANNs represented stimuli in a manner consistent with signal detection theory. Notably, the internal activation distributions observed here were approximately normal; yet, for other task contexts or stimulus classes, deviations from normality could occur, and in such cases provide valuable insight into underlying representational mechanisms or biases. However, the use of ANNs as a platform for evaluating the external-to-internal transformation is likely not limited to an SDT-like framework, and future work could evaluate this transformation within ANNs using other theory-based frameworks, such as the sequential sampling theories (Rafiei et al., 2024).

### Using ANNs as Hypothesis-Generation Platforms

Although we tested the ANNs after observing the human performance, the existence of these similarities suggests that ANNs may sometimes be useful for generating hypotheses about patterns of behavior across stimulus and task conditions. The reasoning here is that some tasks may involve built-in constraints, such that most systems that learn to complete the task, regardless of their details, will also exhibit the same dependencies. We believe that this may be why the simple ANNs used here were able to reproduce, out of the box, the complex qualitative pattern in human data despite these ANNs being so different from human brains. If so, many ANNs may already be useful as hypothesis-generation platforms.

### Differences and Similarities Between CNN and Human Results

One of the big promises of ANN models is that they can function as increasingly more appropriate models of the human visual system (Doerig et al., 2023; Kriegeskorte, 2015). It is clear that current versions of these models differ from human visual perception in many ways (Bowers et al., 2023), which is not surprising given the vast differences between brains and ANNs in both architecture and training. Nevertheless, despite these vast differences, many similarities between ANNs and brains have also been reported (Fung et al., 2025; Kheradpisheh et al., 2016; Kubilius et al., 2019).

In the current study, we found three main differences between the ANNs and the human participants. First, the ANNs exhibited performance that was substantially higher than that of human participants when the same stimuli were used. Second, ANNs show an idiosyncratic peak-and-dip pattern just before they reach a plateau, while human subjects seem to mostly increase until a plateau with no dips. Third, the peak for the ANNs is at around 7 degrees, while the peak for humans is at around 14 degrees.

The difference in the performance peak between the human subjects and the ANNs is particularly interesting as there are several potential mechanisms that may be driving it. First, the ANN’s receptive field size or shape likely differ from that of neurons in the human visual system (Kietzmann et al., 2019). Second, the pooling mechanisms that combine feature responses may differ between biological and artificial systems (Yamins & DiCarlo, 2016). Third, differences in visual experience or learning conditions may contribute to the shift (Henderson & Serences, 2021); specifically here, the fine-tuning regime and training statistics used for the model reflects only a limited portion of natural image statistics across human visual system development. All these mechanisms may contribute to the difference in the location of the peak between ANNs and humans.

At the same time, there were also two important similarities between ANNs and humans. First, both humans and ANNs exhibited a linear increase of *d*′ as a function of tilt for fine orientations. Second, both humans and ANNs exhibited an increase-then-plateau pattern for coarse orientations. As already mentioned, these results are far from trivial as standard models do not predict these patterns a priori (Figure S2). Overall, while the ANNs certainly don’t capture all features of the human data, they do capture some critical details and therefore could usefully serve as hypothesis-generation tools.

### Generalization to Other ANN Architectures

Here we tested how a very simple visual feature, orientation, maps onto internal evidence in the context of a single class of stimuli (Gabor patches). The simplicity of this task makes it superfluous to employ deep networks, such as the ones in most contemporary deep-learning models. However, more complex features, such as ones that allow for natural image classification or person recognition, require deeper and more complex networks, which also have additional advantages over the simple models obtained here. For example, modern vision transformers (ViT) may improve on human prediction by virtue of being more robust to noise-based adversarial attacks (Bai et al., 2021; Maurício et al., 2023), while models that are specifically designed to mimic biological features, such as feedback connections (e.g., CORNET, V1-Net) may be able to better predict neural patterns. While we did not test such models here, we hypothesize that such more complex networks will not substantially change the observed internal-to-external transformation for orientation due to the simple nature of the task.

### Limitations of the Empirical Results and Future Directions

One limitation of this study is that we focused on one stimulus class (orientation), and thus it remains an open question the extent to which these results generalize to other simple stimuli like motion (Green & Pratte, 2022) or color (Poirson & Wandell, 1990), and to complex stimuli like natural objects or scenes (Evans & Treisman, 2005). A second limitation of this study is that it does not consider how attention alters the external-to-internal transformation, and other tasks such as spatial cuing (Yang et al., 2014) or feature-based attention (Liang et al., 2023; Lu & Itti, 2005) may reveal additional effects on the transformation. Indeed, recent work has begun to explore how ANNs can account for long-standing attentional effects (Srivastava et al., 2024), suggesting that ANNs could help predict the internal decisional evidence not just for external stimulus manipulations but also for manipulations of attention. Finally, our work did not incorporate quantitative metrics of human-ANN similarity (Alvarez & Konkle, 2024).

While such quantitative alignment is valuable for benchmarking and cross-model comparison, the primary objective of the present work is not to evaluate specific models or achieve numerical alignment with human data. Rather, we aim to examine whether qualitative patterns in the external-to-internal mapping emerge naturally through ANN training and therefore serve as a platform for relating external sensory features to internal decisional evidence. Indeed, the results suggest that qualitative human-ANN alignment emerges in networks explicitly trained on the orientation discrimination task. However, it remains an open question whether similar alignment could arise in a fully emergent manner, for example, by using a network pretrained on natural images, freezing the core network weights, and training only a linear readout for orientation categorization. In line with this hypothesis, prior work suggests that certain low-level feature representations in networks pretrained on natural images can affect downstream perceptual tests of a given low-level feature like orientation (Henderson & Serences, 2021). Whereas we focus mostly on qualitative alignment between humans and ANNs, going forward, the incorporation of both qualitative and quantitative similarity would further strengthen this framework for future model-human comparisons.

### Conclusion

Whereas previous work has shown that the external-to-internal mapping often varies from one visual domain to another, here we show that the mapping varies drastically across tasks within the visual domain. We further demonstrated that shallow ANNs, trained on the orientation discrimination task, mirrored the pattern of results observed in human subjects without any additional assumptions or training. We took advantage of the fact that, unlike humans, we can “peer inside the black box” of the ANNs. The analysis of the ANNs internal activity suggested that external sensory information is transformed into internal decisional evidence in complex ways that may not be easily intuited from fitting traditional models. Altogether, these results begin to reveal how external sensory information is mapped onto internal decisional evidence. Critically, our findings suggest that ANNs could serve as a powerful tool for this critical external-to-internal transformation.

## ACKNOWLEDGMENTS

We thank Medha Shekhar and Yunxuan Zheng for their help with code review.

## FUNDING INFORMATION

This work was funded by the National Institute of Health (award: R01MH119189) and the Office of Naval Research (award: N00014-20-1-2622) awarded to D. R., and M. L. G. is funded by the National Institute of Health (award: T32EY007092).

## AUTHOR CONTRIBUTIONS

M.L.G.: Conceptualization; Data curation; Investigation; Methodology; Project administration; Software; Writing – original draft; Writing – review & editing. M.H.: Conceptualization; Investigation; Methodology. R.N.D.: Writing –review & editing. D.R.: Conceptualization; Funding acquisition; Investigation; Methodology; Project administration; Resources; Supervision; Validation; Writing – original draft; Writing – review & editing.

## DATA AND CODE AVAILABILITY STATEMENT

The data and analyses code are available at https://osf.io/v6q3d/.

## Supplementary Material


