# Supplementary material for: Using Artificial Neural Networks to Relate External Sensory Features to Internal Decisional Evidence
Source: Open Mind (Camb). 2026 Jan 15;10:29–46. doi: 10.1162/OPMI.a.317 (PMC13053016; doi:10.1162/OPMI.a.317)
Supplement: Supplementary file 1 [file opmi-10-29-s001.pdf]

# Supplementary Results

## Using artificial neural networks to relate external sensory features to internal decisional evidence

Marshall L. Green[1]*, Mingjia Hu[2], Rachel N. Denison[3], Dobromir Rahnev[1]

[1]School of Psychology, Georgia Institute of Technology, Atlanta, GA
[2]Department of Psychology, Indiana University, Bloomington, IN
[3]Department of Psychological and Brain Sciences, Boston University, Boston, MA
*Corresponding author

**Post hoc power analysis**

A power analysis was not conducted to determine the choice of 13 and 11 subjects for Experiments 1 and 2 respectively, and this number of subjects was obtained out of convenience. In the main paper, random effects models were conducted to estimate the function that best describes how external sensory signal strength maps onto internal evidence as measured with sensitivity (d') for each individual. Although there is not a straightforward way to conduct a post hoc power analysis for estimating the random effects, the post hoc power can easily be estimated for the fixed group effects. For the purposes of a post hoc power analysis, we report only the estimated obtained power for the preferred model in each experiment. In Experiment 1, a simple regression model with a single linear term and the intercept fixed to zero ($F(1,77) = 266.6, p < .001, R^2 = .77$) achieved an observed power of .99 for a sample size of 13. In Experiment 2, a multiple regression model ($F(2, 64) = 163.5, p < .001, R^2 = .83$) with linear and logarithmic terms was estimated to have an observed power of .99 for a sample size of 11.

**Sigmoidal psychometric function analysis**

In our main analysis, we used a standard signal detection theory approach to examine how the magnitude of tilt offset is transformed into the internal decisional evidence for identifying left or right orientation. However, a complementary analytical approach is to plot the data as a rate of choosing one stimulus category over the other and fit a sigmoid function. We fit a Gaussian cumulative distribution function (CDF) defined as:

$$L\left(\frac{1}{2}\left[1 + erf\left(\frac{x-\mu}{\sigma\sqrt{2}}\right)\right]\right) + G$$

Where $\mu$ is the mean and $\sigma$ is the standard deviation of the Gaussian which define the midpoint and rate of the function respectively, $\mathrm{L}$ is the lapse rate for rightward orientation defining the supremum of the function, $\mathrm{G}$ is the lapse rate for leftward orientations defining the infimum of the function, and the standard formulation of the error function ($\mathrm{erf}$, Abramowitz & Stegun, 1948).

As expected, the results are consistent with a sigmoid-shaped function when plotted in this manner (Figure S1). The Experiment 1 results show that the proportion of rightward tilt reports for fine-scale variations in tilt follow a sigmoid pattern with a $\mu$ of -.21, $\sigma$ of 1.51, L of .80, and G of .14. The Experiment 2 results also follow a sigmoid pattern with a $\mu$ of .84, $\sigma$ of 2.78, L of .79, and G of .26.

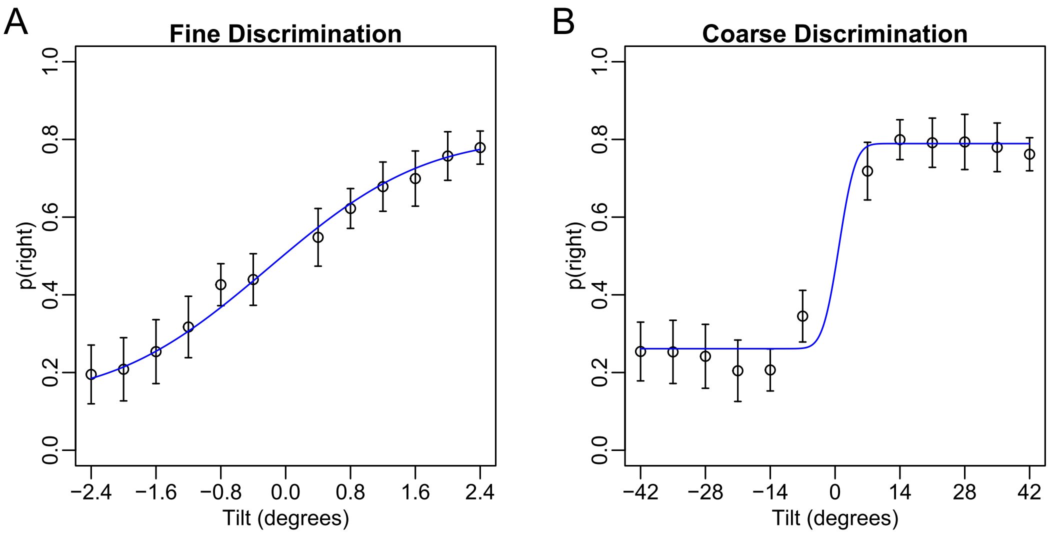


**Figure S1. Proportions of reporting rightward tilt.** (A) Experiment 1 results show that the proportion of rightward tilt reports for fine-scale variations in tilt follow a sigmoid pattern. (B) Experiment 2 results show that the proportion of rightward tilt reports for coarse-scale variations in tilt also follow a sigmoid pattern. Error bars show 95% confidence intervals; lines show best fitting functions.

## Probabilistic Population Coding Model

We examined the predictions of an off-the-shelf probabilistic population coding model for the fine- and coarse-scale orientation discrimination tasks (Figure S2A). Data were simulated from a model based on an encoder-decoder framework (M. L. Green & Pratte, 2022; Jazayeri & Movshon, 2006; Webb et al., 2007) in which evidence for the orientation category of a given stimulus is represented across a bank of orientation selective channels. The orientation sensitivity function of each detector followed a von Mises distribution to ensure that response profiles respected the circular nature of orientation space. The precision of each orientation detector ($\kappa \approx 2.95$) was chosen to approximate the Gaussian half-width half-max of 40°. Each model included 180 motion detectors centered one degree apart. The sensitivity function of the $i^{th}$ orientation detector ($S_i$), which is centered on the orientation $\theta_i$, follows the von Mises density function:

$$S_i(\theta) = \frac{e^{\kappa \cos(\theta - \theta_i)}}{2\pi I_0(\kappa)}$$

The response profile of the $i^{th}$ orientation detector, $D$, to a particular stimulus is given by:

$$R(D) = S_i(\theta) bg$$

where $b$ is the baseline firing rate in spikes per second (10 spike/s) and $g$ is response gain representing the contrast of the Gabor wavelet ($g_s$) and visual noise ($g_n$). The number of spikes ($n_i$) from an orientation detector follows a Poisson distribution with a mean determined by that detector's response profile:

$$Poisson(n_i|D) = e^{-R(D)}\frac{R(D)^{n_i}}{n_i!}$$

The distribution of orientation channel spikes is then multiplied by the log of the channel sensitivity functions to read out the evidence for a given stimulus:

$$logL(\theta) = \sum_{i=-90}^{90} Poisson(n_i|D)\log\left(S_i(\theta)\right)$$

Evidence for whether a given stimulus was left or right of vertical (zero degrees) was computed by taking the argmax of the log Likelihood for orientations greater than zero (rightwards tilts) and less than zero (leftwards tilts). The ratio of evidence for one choice and the other is compared to the criterion of zero.

We examined how the model's orientation sensitivity changes with manipulations of orientation offset. Responses to simulated orientation stimuli were generated from each model 1000 times for each stimulus tilt and contrast. We computed d' using the standard formula (D. M. Green & Swets, 1966) by treating clockwise tilt stimuli as the target and calculating the hit rate (HR) and false alarm rate (FAR). The PPC model predicts that increasing orientation offset in fine-scale increments results in a linear increase in sensitivity (d'), a pattern that is identical to that of human subjects (Figure S2B). Critically, the PPC model also predicts that increasing orientation offset in coarse-scale increments similarly results in a linear increase in sensitivity (d'), a pattern which is starkly different from that of human subjects (Figure S2C). Although this off-the-shelf PPC model does not predict the same pattern of results obtained from human subjects, it is of course likely that a better fit can be obtained if additional assumptions are included in the model.

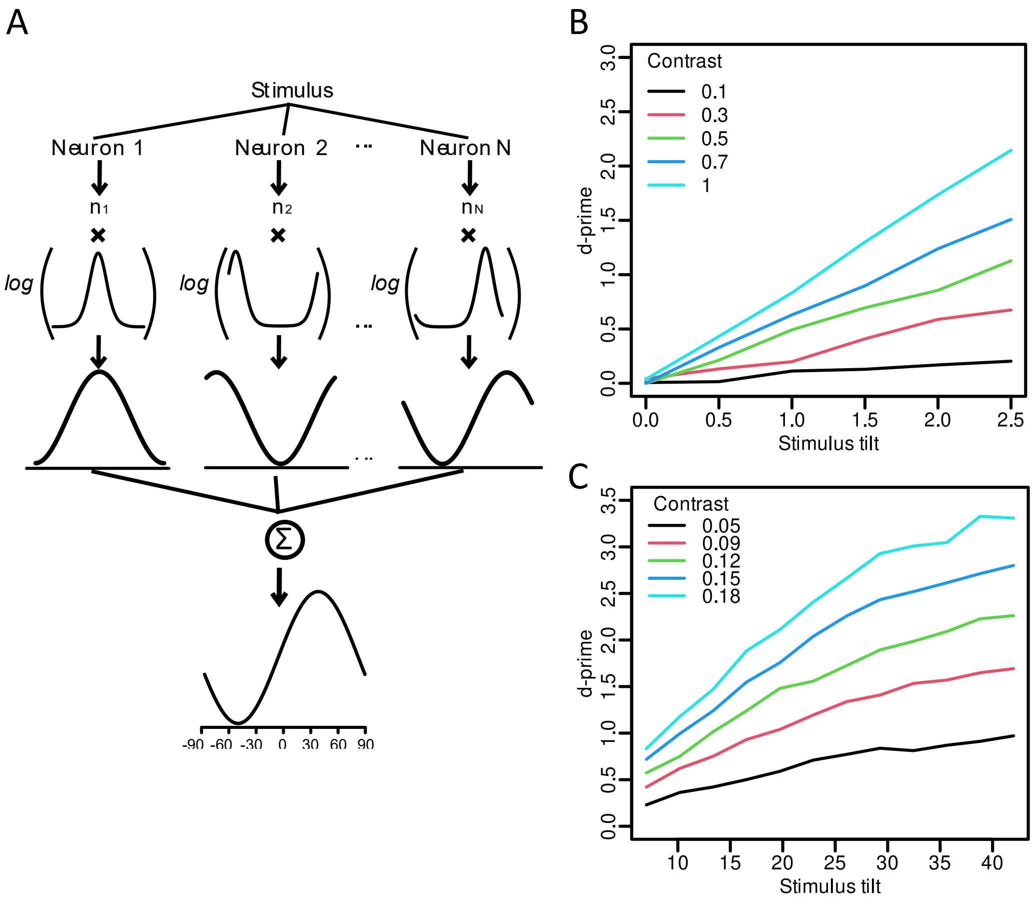


**Figure S2. Predictions from a standard Probabilistic Population Coding (PPC) model**. (A) An off-the-shelf probabilistic population coding model represents orientation evidence as a log likelihood function. (B) The PPC model predicts that increasing orientation offset in fine-scale increments results in a linear increase in sensitivity (d'). (C) The PPC model similarly predicts that increasing orientation offset in coarse-scale increments results in a linear increase in sensitivity.

## Artificial neural network contrast experiment

In the main paper, we reported the results of testing the trained ANNs on fine and coarse-scale orientation categorization tasks with contrast varying across five levels ranging from 2.5% to 8.5%, and visual noise fixed at 100%. The goal of that test was to examine the effect of tilt magnitude and stimulus contrast on the external-to-internal transformation (Figure 5). However, this raises a question of whether this particular range of stimulus parameter values reflect the differences between the empirical results for the high-contrast stimuli of Experiment 1 and the low-contrast, high-noise stimuli of Experiment 2. To address this question, we examined ANN performance at noise levels of 25%, 50% and 75%. The procedure was otherwise the same (see Methods). The results of manipulating noise replicated the experimental manipulation of stimulus contrast: all three ANN models reproduced both the linear relationship between d' and orientation for fine-grained tilts that we observed in Experiment 1 (Figure S3A,C,E), and the non-linear, flat relationship between d' and orientation for coarse-grained tilts that we observed in Experiment 2 (Figure S3B,D,F). The number of layers in the ANN models again had no effect on the pattern of

results. Overall, the amount of noise had a scaling effect on the function for both the fine- and coarse-scale tasks but did not fundamentally change the shape of the function that describes the underlying external-to-internal transformation.

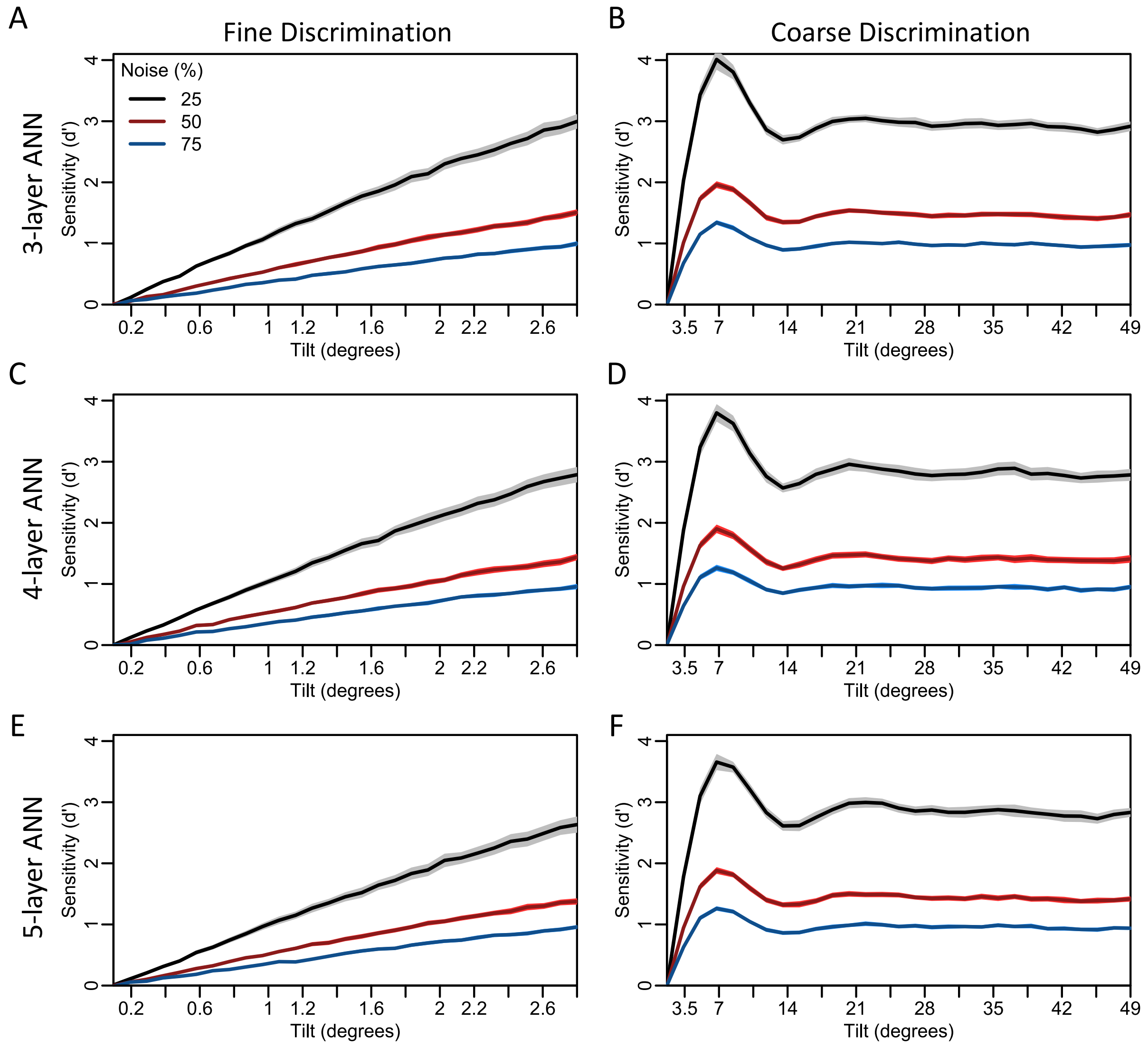


**Figure S3. ANN results with lower noise levels.** In the main paper, we showed results of testing ANNs with stimuli that had noise level of 100%. Here, we examined ANN performance at noise levels of 25%, 50% and 75%. The ANNs results (lines) again reproduced the empirical results from Experiments 1 and 2 – fine-scale increments in the tilt magnitude from 0 to 2.8 degrees were linearly transformed into internal evidence (A,C,E), but coarse-scale increments in the tilt magnitude up to 49 degrees were nonlinearly transformed into internal evidence (B,D,F). The performance of the 3-layer (A,B), 4-layer (C,D) and 5-layer (E,F) models was nearly identical. For both tasks, the stimulus contrast was maintained at 2.5% and increasing the contrast of the visual noise had a scaling-up effect on sensitivity but had little effect on the general trend in the relationship between sensitivity and tilt magnitude. The shaded regions show 95% confidence intervals, but they are difficult to see in the figure due to the small intervals and the Y-axis range.

**Representational similarity analysis**

We analyzed the representational similarity (Diedrichsen & Kriegeskorte, 2017) of the final fully connected layer of each model to examine whether the models were representing the stimuli in a manner consistent with the categorization task. That is, we expect that the representational similarity analysis (RSA) would indicate a clear categorical difference in the way that leftward and rightward stimuli were represented. Similarly, we expect that stimuli with more similar orientations would be represented more similarly than less similar orientations, and therefore in addition to the clear categorical boundary between left and right stimuli the similarity between any one orientation and all other orientations should decrease in a graded manner as orientation is varied systematically. We extracted the activations of the last fully connected layer for each ANN in response to 1,000 Gabor stimuli presented at 5.25% contrast and 100% noise for tilts ranging from 0 to 49 degrees were extracted and averaged. The average activation for each tilt was correlated with the activation for every other tilt to generate the representational dissimilarity matrices (RDMs). The results of the RSA applied to the 3-layer ANN demonstrated a clear categorical difference in the way that leftward (negative) and rightward (positive) tilts were represented, as indicated by greater correlations within category and lesser correlations between categories (Figure S4A). The 3-layer ANN additionally exhibited a gradation of similarity as a function of tilt such that more similar tilts exhibited a greater correlation than less similar tilts. Both the 4-layer (Figure S4B) and 5-layer (Figure S4B) ANNs exhibited similar patterns of results, though the differences in the absolute correlations increased with model depth. These results are congruent with our SDT-like analysis on the linear output from the final layer in which signal and noise distributions were more similar leading to less discriminability. However, the RSA results also revealed a robust increase in representational similarity for tilts beyond 7 degrees, as demonstrated by the blocked patterns. This pattern is consistent with our finding that discriminability plateaus for sufficiently large tilts.

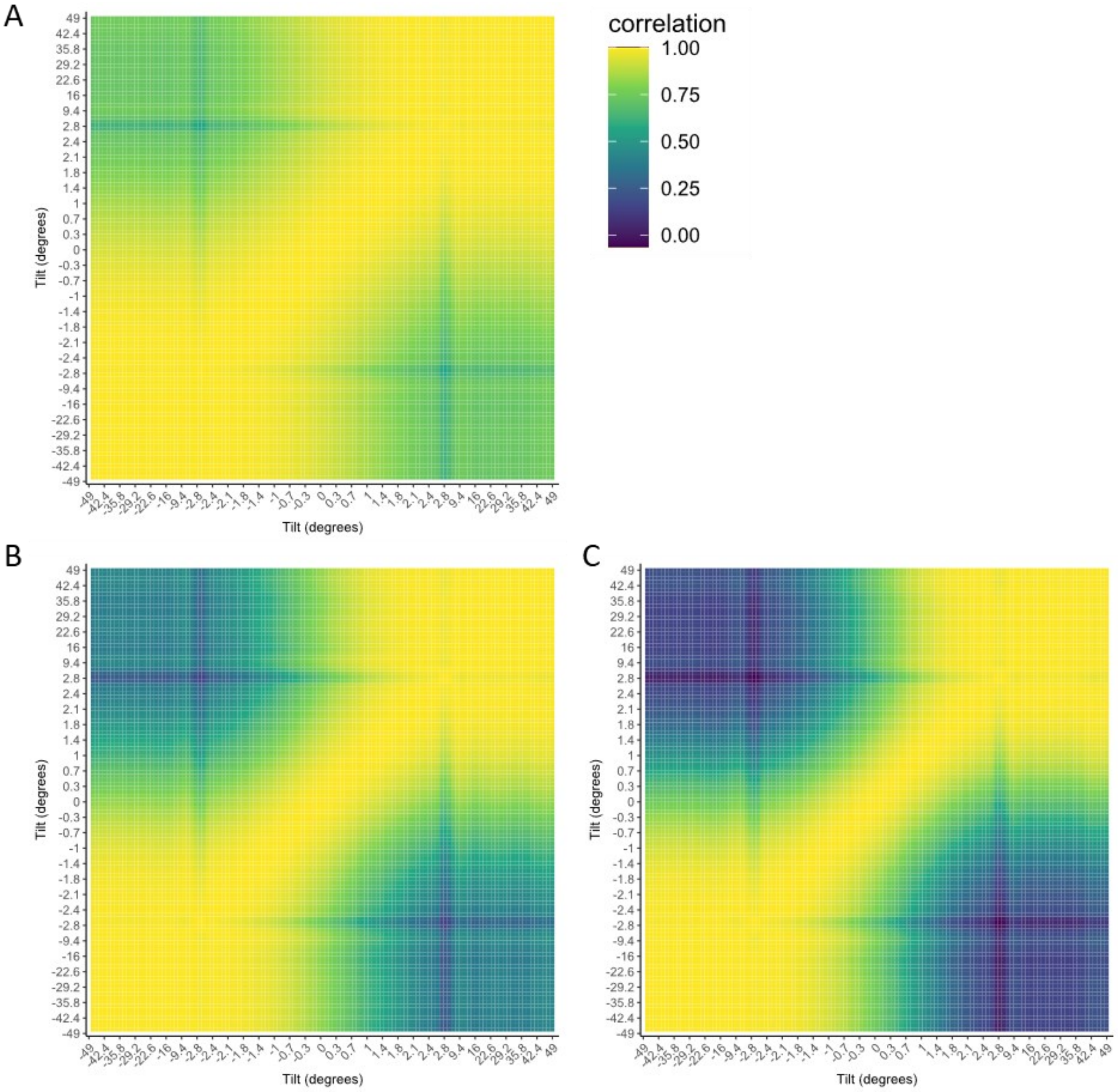


**Figure S4. Representational dissimilarity matrices (RDMs).** The activations of the last fully connected layer for each ANN in response to 1,000 Gabor stimuli presented at 5.25% contrast and 100% noise for tilts ranging from 0 to 49 degrees were extracted and averaged. The average activation for each tilt was correlated with the activation for every other tilt to generate the RDMs. (A) The 3-layer ANN demonstrated a clear categorical difference in the way that leftward (negative) and rightward (positive) tilts were represented, as indicated by greater correlations within category and lesser correlations between categories. The ANN also demonstrated a gradation of similarity as a function of tilt such that more similar tilts exhibited a greater correlation than less similar tilts. Both the (B) 4-layer and (C) 5-layer ANNs exhibited similar patterns of results, though the differences in the absolute correlations increased with model depth.

## References

Abramowitz, M., & Stegun, I. A. (1948). *Handbook of mathematical functions with formulas, graphs, and mathematical tables* (Vol. 55). US Government printing office.

Green, D. M., & Swets, J. A. (1966). *Signal detection theory and psychophysics* (Vol. 1). Wiley.

Green, M. L., & Pratte, M. S. (2022). Local motion pooling is continuous, global motion perception is discrete. *Journal of Experimental Psychology: Human Perception and Performance*, *48*(1), 52–63. https://doi.org/10.1037/xhp0000971

Jazayeri, M., & Movshon, J. A. (2006). Optimal representation of sensory information by neural populations. *Nature Neuroscience*, *9*(5), 690–696. https://doi.org/10.1038/nn1691

Webb, B. S., Ledgeway, T., & McGraw, P. V. (2007). Cortical pooling algorithms for judging global motion direction. *Proceedings of the National Academy of Sciences*, *104*(9), 3532–3537. https://doi.org/10.1073/pnas.0611288104
